# Receiving Palliative Treatment Moderates the Effect of Age and Gender on Demoralization in Patients with Cancer

**DOI:** 10.1371/journal.pone.0059417

**Published:** 2013-03-15

**Authors:** Sigrun Vehling, Karin Oechsle, Uwe Koch, Anja Mehnert

**Affiliations:** 1 Department of Medical Psychology, University Medical Center Hamburg-Eppendorf, Hamburg, Germany; 2 Department of Oncology/Hematology/Pneumology, University Medical Center Hamburg-Eppendorf, Hamburg, Germany; 3 Deanery, University Medical Center Hamburg-Eppendorf, Hamburg, Germany; 4 Department of Medical Psychology and Medical Sociology, Division of Psychosocial Oncology, University Medical Center Leipzig, Leipzig, Germany; The University of Hong Kong, Hong Kong

## Abstract

**Background:**

Existential distress is an important factor affecting psychological well-being in cancer patients. We studied occurrence and predictors of demoralization, a syndrome of existential distress, in particular the interaction of age, gender, and curative vs. palliative treatment phase.

**Methods:**

A cross-sectional sample of N = 750 patients with different tumor sites was recruited from in- and outpatient treatment facilities. Patients completed the following self-report questionnaires: Demoralization Scale, Patient Health Questionnaire-9, Illness-Specific Social Support Scale Short Version-8, and physical problems list of the NCCN Distress Thermometer. Moderated multiple regression analyses were conducted.

**Results:**

We found high demoralization in 15% and moderate demoralization in 8% of the sample. Curative vs. palliative treatment phase moderated the impact of age and gender on demoralization (three-way interaction: b = 1.30, P = .02): the effect of age on demoralization was negative for women receiving palliative treatment (b = −.26, P = .02) and positive for men receiving palliative treatment (b = .25, P = .03). Effects of age and gender were not significant among patients receiving curative treatment. Female gender was associated with higher demoralization among younger patients receiving palliative treatment only. Analyses were controlled for significant effects of the number of physical problems (b = 6.10, P<.001) and social support (b = −3.17, P<.001).

**Conclusions:**

Existential distress in terms of demoralization is a relevant problem within the spectrum of cancer-related distress. It is associated with a complex interaction of demographic and medical patient characteristics; existential challenges related to palliative treatment may exacerbate the impact of age- and gender-related vulnerability factors on demoralization. Psychosocial interventions should acknowledge this interaction in order to address the individual nature of existential distress in subgroups of cancer patients.

## Introduction

Adequate management of existential distress is essential to psychosocial care in cancer, yet it has only recently been focused in specialized interventions and distress measures [1]–[3]. Existential distress in cancer patients may arise from the impact of multiple existential challenges raised by cancer diagnosis and treatment, which include fear of death and dying and the threat to fundamental human needs for autonomy, self-worth, relatedness, and meaning [4], [5]. Evidence grows showing that existential distress is a relevant factor influencing psychological well-being in cancer, although heterogeneous conceptualizations have been used [6]. However, few studies have examined the occurrence of existential distress and predicting factors, mostly limited to small samples of patients with advanced illness.

The concept of demoralization provides a profound base for assessment of existential distress in cancer patients not covered by standard diagnostic approaches. Clarke and Kissane [7] define demoralization as an affective state of loss of meaning and hopelessness, with cognitions of helplessness and personal failure, subjective incompetence, and social alienation. Research has repeatedly applied this concept to severe medical illness, emphasizing that demoralization is essentially characterized by an entrapped feeling that “nothing can be done” and a subsequent loss of hope and meaning, while the two core symptoms of major depression, anhedonia and loss of interest, are typically not present [8]–[11]. Factor analytic studies support the conceptual and clinical separation of demoralization and major depression in cancer patients [12], [13]. Validated measures have shown good psychometric properties and capacity to differentiate demoralized from depressed patients, despite considerable overlap [14], [15].

Previous studies examining predictors of demoralization have found positive associations with the number of physical problems and negative associations with social support and partnership [12], [16]–[18]. Results regarding age, gender, and treatment phase are inconsistent, however. Positive [16], negative [12], [17], [19], or no associations with age [15], [20] were found. Most studies found no gender differences [12], [15], [16], [20], except for one study [17]. In addition, while it is widely accepted that the existential burden among patients with advanced cancer is linked to existential distress [6], [21], [22], palliative vs. curative treatment phase or advanced vs. early disease stage were not associated with higher demoralization [15], [16], [20], [23]. These studies did mostly not control for confounding variables and none of them considered interaction effects. More complex underlying associations may however account for absent effects of cancer-related variables [24]. Hence, combined with the pattern of previous results, a three-way interaction of age, gender, and treatment phase may contribute to inconsistent associations between these factors and demoralization in previous studies.

The aims of the present study were to (1) assess the occurrence of demoralization in a large sample of cancer patients with mixed tumor sites, and (2) examine the impact of the age×gender×treatment phase three-way interaction on demoralization, controlling for the number of physical problems and positive social support. We (3) examined if the impact of these predictors on demoralization was independent of the overlap between demoralization and depression.

We hypothesized a significant positive effect of physical problems and a significant negative effect of positive social support on demoralization (a). Further, a significant age×gender×treatment phase interaction under control of physical problems and social support was assumed (b). We finally hypothesized a significant effect of the number of physical problems, positive social support, and the age×gender×treatment phase interaction on demoralization when depression was controlled (c).

## Methods

### Ethics Statement

The study was approved by the local medical association research ethics committee (Ethik-Kommission der Ärztekammer Hamburg). Written informed consent was obtained from all participating patients.

### Participants

**Figure 1:**
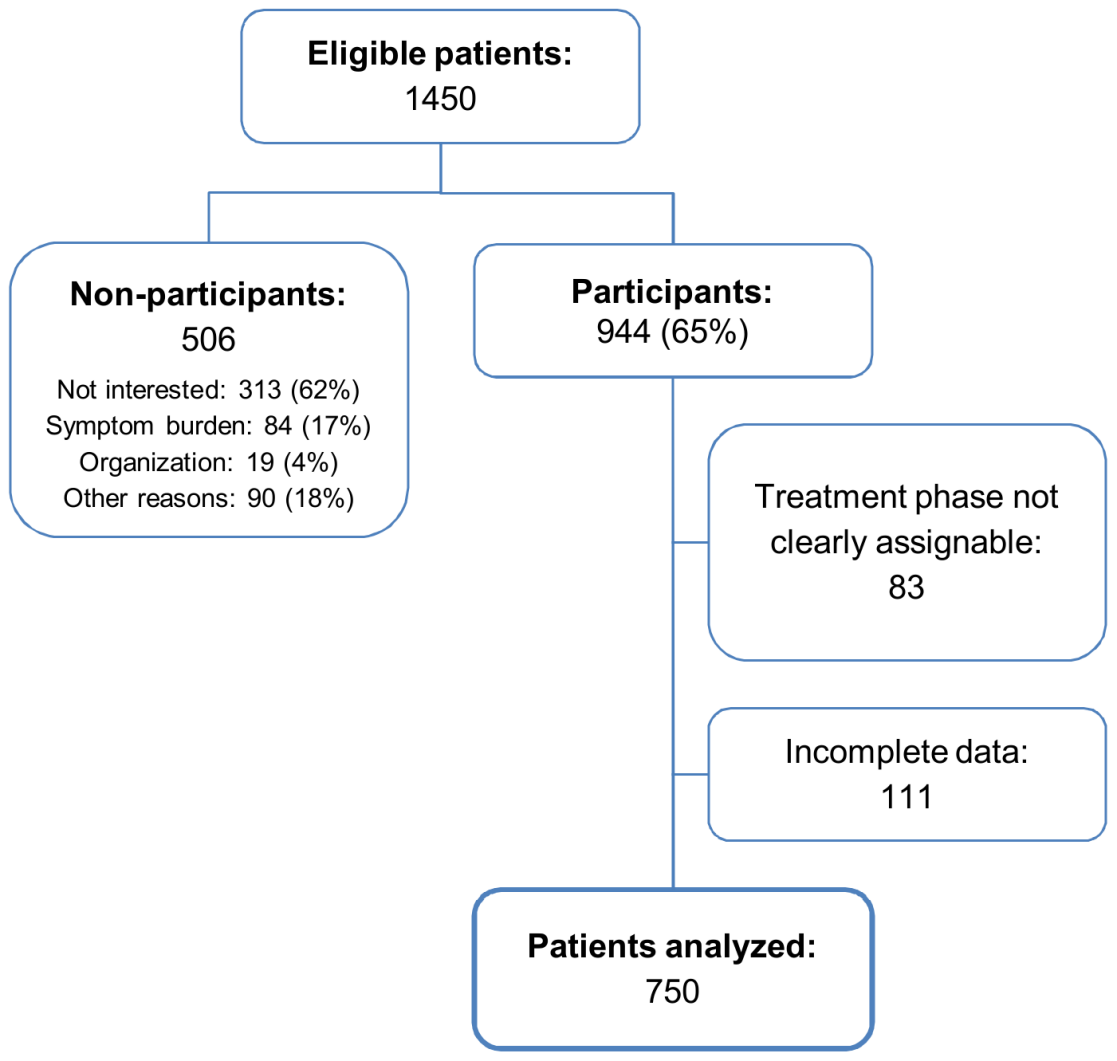
Recruitment of study sample.

A subsample of a representative multicenter epidemiologic study on the prevalence of comorbid psychiatric disorders [25] involving patients recruited from the major study center in Hamburg, Germany was analyzed. Patients with malignant tumors aged 18 to 75 years were consecutively recruited from acute care hospitals, outpatient treatment facilities and rehabilitation centers between March 2009 and October 2010 by trained research assistants. Exclusion criteria were severe physical and cognitive impairment and insufficient proficiency in German to provide informed consent and complete questionnaires. Patients were informed about the study and provided questionnaires and stamped return envelopes after written informed consent had been obtained. Basic demographic data was recorded from non-participants. Non-participants were slightly older than participants (M = 60.5, SD = 10.6 vs. M = 57.7, SD = 12.1; P<.001), but did not differ in gender (P = .10). Most frequent reasons to decline participation were lack of interest (62%) and physical or psychological symptom burden (16%). Patient flow is shown in figure 1.

### Measures

Sociodemographic data including age, gender, marital status, and education were assessed by a standardized self-report questionnaire. Medical data including tumor entity, date of first diagnosis, UICC-stage, and curative vs. palliative treatment phase were obtained from medical charts and professional treatment reports. Classification of treatment phase was based on the current intention of oncologic treatment as evaluated by the attending physician. This evaluation was based on tumor stage (presence and extent of metastases), disease progression (remission, stable disease, or progressive disease), and current and past oncologic treatment modalities (type and dose of chemotherapy, radiotherapy, surgery, and other therapies).

*Demoralization* within the past two weeks was assessed by the German version of the Demoralization Scale (DS) [14], [17]. Total scores may range from 0 to 96. A cutoff score of 36 was used to indicate high demoralization and a cutoff score of 30 was used to indicate moderate demoralization [14], [16]. The *number of physical problems* was measured by the physical problems list of the NCCN Distress Thermometer [26], assessing the presence of 21 common physical symptoms in cancer patients. *Social support* was measured by the positive support subscale of the Illness-Specific Social Support Scale Short Version-8 (ISSS-8) [27], [28]. Total scores may range from 0 to 16. *Depression* within the past two weeks was assessed using the DSM-IV-based depression module of the Patient Health Questionnaire (PHQ-9) [29]. Scores may range from 0 to 27. A cutoff score of 10 was used to indicate moderate depression and a cutoff score of 15 was used to indicate high depression.

### Statistical Analysis

Descriptive statistics including means, standard deviations, and frequencies of demoralization were calculated across sample characteristics. Group differences and bivariate associations were calculated using ANOVA, t-test, χ^2^-test, and Pearson correlation coefficient. Hierarchical moderated multiple regression analysis was used to analyze our predictor and interaction hypotheses concerning demoralization. Applying the Aiken and West [30] procedure for probing three-way interactions, predictor variables were entered into the regression equation in three subsequent steps. In the first step (model 1), number of physical problems, social support, age, gender, and treatment phase were simultaneously entered, estimating first-order effects of all variables. In the second step (model 2), the two-way-interactions age×gender, age×treatment phase, and gender×treatment phase were entered. The three-way interaction age×gender×treatment phase was entered separately in the third step (model 3). The fourth step included depression as a control variable in order to examine if hypothesized predictors showed a significant effect over and above the effect of depression on demoralization (model 4).

For regression analyses, unstandardized regression coefficients (b-values) are reported only, as standardized coefficients of interaction terms are not interpretable [30], [31]. Continuous predictor variables were standardized and categorical variables were contrast coded prior to analyses. Thus, in presence of a significant interaction effect, the simple effect of each predictor variable involved in the interaction term is the effect of that variable at the average level (i.e. zero) of the other variables [30], [32]. Interaction terms were calculated by multiplication of respective variables.

The following analyses were carried out to further analyze the possible three-way interaction: simple slopes of the relationship between age and demoralization were calculated for all gender×treatment phase subgroups (i.e., female/palliative treatment, male/palliative treatment, female/curative treatment, and male/curative treatment) [30]. Further, differences between each pair of simple slopes were tested for significance using the Dawson and Richter [33] slope difference test. Power calculation according to the Dawson and Richter equation showed that our sample size was sufficient to detect a slope difference of small effect size (i.e., Δb = 0.1) with power of β = 0.8. Error variances were homogenous across gender×treatment phase subgroups, with error variance ratios ranging between 1:1.01 and 1:1.36 [34], [35]. All p-values are two-sided, and all analyses were performed using R version 2.14.1 and PASW Statistics version 18.0.

## Results

### Sample Characteristics and Frequency of Demoralization

Table 1 shows demographic and medical sample characteristics and demoralization means, standard deviations, and frequencies across subgroups. Of the total sample, 15.3% were seriously demoralized (DS≥36) and 8.4% were moderately demoralized (DS≥30). The mean demoralization score was 20.8 (SD = 13.9, range = 0–69).

**Table 1:** Sample characteristics and descriptive statistics for demoralization.

	Sample		Demoralization Mean			Demoralization Frequency (DS≥36)		
Variable	N	(%)	M	(SD)	P^a^ (d/η^2^)	N	(%)	P^b^ (φ)
Total sample	750	100	20.8	13.9		115	15.3	
*Gender*					<.001 (.028)			.001 (.123)
Female	394	52.5	23.1	14.7		77	19.5	
Male	356	47.5	18.4	12.6		38	10.7	
*Age, mean (SD)*	57.7 (12.2)				<.001 (.034)			<.001 (.167)
18–39	65	8.7	25.4	16.2		18	27.7	
40–59	313	41.7	22.9	14.8		61	19.5	
60–75	372	49.6	18.4	12.2		36	9.7	
*Marital status*					.001 (.024)			.10 (.092)
Married/partnership	587	79.6	20.0	13.7		85	14.5	
Single	67	9.1	25.6	15.2		14	20.9	
Separated/divorced	54	7.3	24.9	13.5		11	20.4	
Widowed	29	3.9	16.7	8.8		1	3.4	
*Education*					.11 (.008)			.20 (.079)
Elementary school (8–9 years)	174	23.4	19.0	12.4		21	12.1	
Junior high school (10 years)	241	32.4	20.9	14.9		35	14.5	
High school (13 years)	113	15.2	23.1	15.1		24	21.2	
University	216	29.0	21.1	13.3		35	16.2	
*Tumor site*					<.001 (.035)			.015 (.152)
Breast	228	30.4	21.2	14.7		38	16.7	
Prostate	198	26.4	17.0	12.0		15	7.6	
Hematologic	59	7.9	22.4	13.5		12	20.3	
Gynecologic	58	7.7	25.1	14.4		13	22.4	
Colorectal	40	5.3	19.6	11.5		4	10.0	
Lung	29	3.9	23.9	13.6		6	20.7	
Head and Neck	27	3.6	22.7	15.6		7	25.9	
Other	111	14.8	23.1	14.7		20	18.0	
*Treatment phase*					.032 (.006)			.27 (.041)
Curative	584	77.9	20.3	13.7		85	14.6	
Palliative	166	22.1	22.9	14.4		30	18.1	
*UICC tumor stage*					.30 (.002)			.52 (.024)
0-II	419	60.9	20.7	13.9		64	15.3	
III–IV	269	39.1	21.8	14.1		46	17.1	
*Months since initial diagnosis, mean (SD)*	27.5 (47.7)							
*Type of disease*					.001 (.019)			.056 (.088)
Initial diagnosis	577	76.9	19.9	13.6		79	13.7	
Recurrence	117	15.6	25.2	14.5		26	22.2	
Second tumor	56	7.5	21.1	14.3		10	17.9	
*Treatment setting*					.23 (.002)			.042 (.076)
Inpatient	354	47.2	20.2	13.3		44	12.4	
Outpatient	396	52.8	21.4	14.4		71	17.9	
*No. of physical problems, mean (SD)*	5.4 (3.8)							
*Social support, mean (SD)*	13.8 (2.7)							
*Depression, mean (SD)*	5.9 (4.6)				<.001 (.252)			<.001 (.427)
Low	589	79.3	17.3	11.3		45	7.6	
Moderate (PHQ≥10)	111	14.9	31.8	14.5		43	38.7	
High (PHQ≥15)	43	5.8	39.6	14.3		25	58.1	

Abbreviations: DS, demoralization; d, t-test effect size; η^2^, ANOVA effect size; φ, χ^2^-test effect size; SD, standard deviation; PHQ, Patient Health Questionnaire.

a [t-test/ANOVA], ^b^[χ^2^-test].

### Predictors of Demoralization

Bivariate correlations with demoralization and intercorrelations among predictors are reported in table 2. Predictor intercorrelations were small to moderate and variance inflation factors were below 1.8 for all coefficients, showing no evidence of multicollinearity.

**Table 2:** Bivariate correlations with demoralization and intercorrelations among predictors.

Predictors	Demoralization		No. of physical problems		Social support		Age		Gender#		Treatment phase§	
	r	P	r	P	r	P	r	P	r	P	r	P
No. of physical problems	.49	<.001	–	–								
Social support	−.28	<.001	−.12	.002	–	–						
Age	−.16	<.001	−.20	<.001	.06	.10	–	–				
Gender#	−.17	<.001	−.17	<.001	.01	.71	.32	<.001	–	–		
Treatment phase§	.08	.032	.16	<.001	−.02	.54	.04	.29	−.02	.62	–	–
Depression	.61	<.001	.62	<.001	−.11	.002	−.27	<.001	−.24	<.001	.11	.003

# −1  =  female, +1  =  male.

§ −1  =  curative, +1  =  palliative.

Results of moderated regression analyses are shown in table 3; model 3 shows coefficients resulting after inclusion of all interaction terms. A higher number of physical problems (b = 6.10, P<.001) and lower social support (b = −3.17, P<.001) were significant predictors of demoralization (hypothesis a). Analyses further showed a small but significant three-way interaction between age, gender, and treatment phase (b = 1.30, P = .018, ΔR^2^ = .006) (hypothesis b).

**Table 3:** Hierarchical regression analyses of predictors and interaction effects contributing to demoralization.

	Demoralization											
	Model 1			Model 2			Model 3			Model 4		
Predictors	b	SE	P	b	SE	P	b	SE	P	b	SE	P
No. of physical problems*	6.15	.45	<.001	6.19	.45	<.001	6.10	.45	<.001	2.35	.49	<.001
Social support*	−3.08	.43	<.001	−3.16	.43	<.001	−3.17	.43	<.001	−2.81	.39	<.001
*Simple effects*												
Age*	−.46	.46	.32	−.30	.55	.59	−.33	.55	.54	.40	.50	.42
Gender#	−1.09	.46	.017	−1.64	.53	.002	−1.71	.53	.001	−1.03	.48	.031
Treatment phase§	.06	.52	.91	.12	.53	.83	−.09	.53	.87	−.13	.48	.78
*Two-way interactions*												
Age × gender				1.11	.46	.015	1.81	.54	.001	1.30	.49	.009
Age × treatment phase				.15	.55	.78	.23	.55	.67	.12	.50	.81
Gender × treatment phase				−.95	.53	.072	−1.05	.53	.048	−.99	.48	.039
*Three-way interaction*												
Age × gender × treatment phase							1.30	.55	.018	.85	.49	.086
*Control variable*												
Depression*										6.62	.50	<.001
*Explained variance*												
ΔR^2^				.008			.006			.131		
R^2^	.295			.303			.309			.440		
Adjusted R^2^	.290			.296			.300			.432		

Abbreviations: b, regression coefficient (unstandardized); SE, standard error of b; ΔR^2^, change in R^2^ compared to previous model.

* Standardized predictors.

# −1 = female, +1 = male.

§ −1 = curative, +1 = palliative.

**Figure 2:**
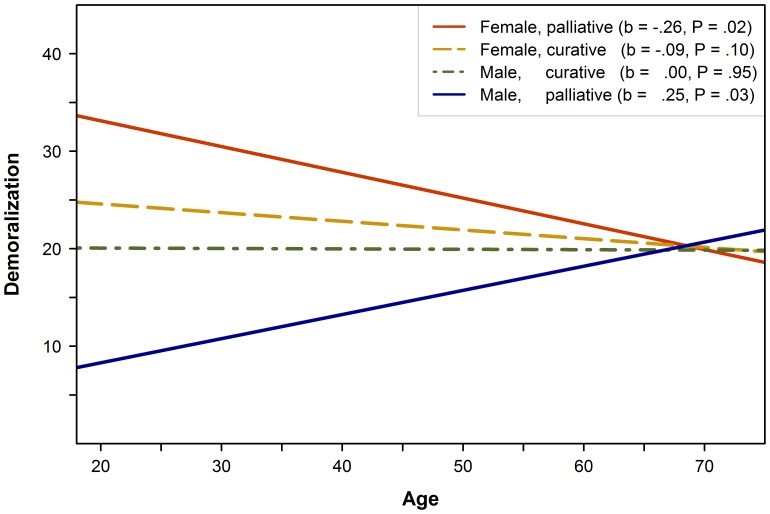
Three-way interaction of age, gender, and treatment phase on demoralization. Simple slopes of the relationship between age and demoralization for gender×treatment phase subgroups. Analyses were controlled for the number of physical problems and social support, i.e. simple slopes were calculated for sample means of these variables. Abbreviations: b, regression coefficient of simple slope (unstandardized).

The plot of the three-way interaction effect in figure 2 shows simple slopes of relationships between age and demoralization for all gender×treatment phase subgroups, controlling for physical problems and social support. Simple slope analyses showed a significant negative relationship between age and demoralization for women receiving palliative treatment, and a significant positive relationship for men receiving palliative treatment. There was no significant association with age for both men and women receiving curative treatment.

As shown in table 4, there was a significant difference between the simple slopes of the regression lines for men receiving palliative treatment and women receiving palliative treatment (t = 3.41, P = .001).

**Table 4:** Test of differences between simple slopes of the relationship between age and demoralization.

	(1)		(2)		(3)		(4)	
Simple slope	Female, palliative		Female, curative		Male, curative		Male, palliative	
	(b = −.26, SE = .11, P = .017)		(b = .09, SE = .06, P = .10)		(b = .00, SE = .06, P = .95)		(b = .25, SE = .11, P = .028)	
	t	P	t	P	t	P	t	P
(1) Female, palliative	–	–						
(2) Female, curative	−1.47	.14	–	–				
(3) Male, curative	1.43	.15	0.02	.98	–	–		
(4) Male, palliative	3.41	.001	1.97	.049	1.89	.060	–	–

Abbreviations: b, regression coefficient of simple slope (unstandardized); SE, standard error of b; t, t-value calculated by Dawson & Richter slope difference test.

Examining gender differences, simple slope analyses further showed that demoralization was higher in women for patients receiving palliative treatment aged 61 or younger (b_61_ = 1.92, P = .40). There were no gender differences among patients receiving curative treatment or patients older than 61. Palliative treatment phase was associated with higher demoralization in women and with lower demoralization in men for patients aged 51 or younger (women: b_51_ = 1.98, P = .045; men: b_51_ = −1.55, P = .049). The small additional significant simple effect of gender on demoralization (table 3, model 3) indicates higher demoralization in women than in men at (hypothetical) average sample levels of age and treatment phase.

Model 4 finally shows regression results after including depression as a control variable (table 3). When depression was controlled, b-coefficients were lower for all significant predictors. All b-coefficients were still significant, except for the interaction (P = .086). Thus hypothesis (c) received only partial support.

## Discussion

We found moderate to high demoralization in 24% of our sample including cancer patients with mixed tumor sites receiving curative and palliative oncologic treatment. Demoralization was associated with a higher number of physical problems, less positive social support, and a three-way interaction of age, gender, and curative vs. palliative treatment phase in a controlled model. Effects were not statistically explainable by the overlap between demoralization and depression. The interaction identified different relationships between age and demoralization depending on the combination of gender and treatment phase. Demoralization decreased with age only among women receiving palliative treatment and increased with age only among men receiving palliative treatment. Demoralization was not significantly related to age in curative patients.

Previous studies have, to our knowledge, rarely examined interactions of demographic and medical variables on demoralization or related outcomes in cancer. Our results are yet comparable to previous literature to the extent to which studies have examined simple effects of age, gender, or treatment phase in homogeneous samples, controlled for respective intercorrelations, and controlled for predictors that covary with these variables. Our results are consistent with the limited effect of curative vs. palliative treatment on demoralization in our earlier study [16]. The higher percentage of men receiving palliative treatment (19% vs. 10%) may have contributed to the positive effect of age in that study. Similarly, Jones *et al.* [36] found no effect of advanced tumor stage on hopelessness in a mixed sample, and no associations between age and demoralization were found in samples including a high rate of early-stage patients [15], [20]. More generally, our results are consistent with reviews concluding a negative effect of age on distress in breast and gynecological cancer [37], [38], and missing [39], [40] or positive [41] age effects in prostate cancer. Interestingly, Miller *et al.* [42] found no gender differences in patients with metastasized lung and gastrointestinal cancer. Our findings support the idea that the higher age of patients with these tumors is a possible explanation to this result. In line with our findings, a recent prospective study showed an age×gender interaction indicating improvement in distress among younger men and older women, but not among older men and younger women [43].

Despite the limited overall impact of palliative treatment on existential distress, our results indicate that related existential challenges moderate the impact of age- and gender-specific vulnerability factors on demoralization in cancer. Younger age has been linked to a number of vulnerability factors, including cancer as an “off-time” event, greater interference with role functioning, and less coping experience from negative life events [44]. Higher distress in female patients has been related to gender roles and socialization effects as most relevant causal vulnerability factors [45], [46]. Our findings suggest that these factors and their interaction may become more relevant in face of a high existential burden, which, given our analyses controlled for the number of physical problems, is not entirely captured by physical impairment and its consequences. But what is the nature of these existential challenges and how can age- and gender-related differences in responses to them be explained?

Rodin and Zimmerman [4] suggest that an existential challenge essential to psychological well-being for patients with advanced cancer is the capacity to tolerate the “double awareness” of realizing death and yet maintaining a sense of meaning from sources of personal value. While this challenge may generally raise more difficulties for younger patients, our results indicate that related coping responses may diverge across gender. For younger women, awareness of a limited prognosis may more often imply an inability to shift focus toward new sources of hope and meaning, increasing the risk for demoralization. On the other hand, younger men may more often distract awareness from the reality of a foreshortened life and focus on sustaining a sense of normality and control, causing them to report lower levels of demoralization. This idea is consistent with the more frequent use of ruminative coping in response to negative events in women [47]. Among younger women with breast cancer, worries about family members were further found to be a central source of distress [48], and to impede coping with the existential dialectic of hope and hopelessness in the terminally ill [49]. On the other hand, male gender role expectations of stoicism and self-reliance have been suggested to explain distracting coping responses in men [50]. Evidence concerning gender differences in coping is nevertheless limited, and information is especially missing about emotion regulation in men [47]. Similarly, there is a lack of studies investigating psychosocial adjustment to existential challenges among younger men with advanced cancer.

We further found that gender differences decreased with higher age. This is consistent with an age-related increase of protective existential factors in both genders: spiritual well-being and attachment security were shown to mediate the decrease of depression with age [51], and negative associations were found between personal meaning, sense of coherence, spiritual well-being, or attachment security and demoralization [23], [52], hopelessness [53], and loss of dignity [21].

The rate of 24% reporting at least moderate demoralization in the present study underscores the relevance of demoralization within the spectrum of distress related to cancer. Associations between independent variables and demoralization were not simply accounted for by depression as an explanatory third variable, supporting the idea that demoralization covers a distinct domain of existential distress experienced by cancer patients. Longitudinal studies however need to confirm that prolonged and untreated existential distress is an important factor in development of depression and the desire for hastened death [53], [54], while first results point toward this direction [55]. The strong negative effect of positive social support in our study further strengthens the good response of demoralization to supportive psychosocial interventions drawn from clinical experience [9], [56]. The association with positive support was stronger for demoralization compared to depression, strengthening the rationale of interventions directly focusing on existential concerns.

Limitations of our study include the number of non-participants due to high physical and psychological symptom burden, which might have led to an underestimation of demoralization in our sample, especially in older patients, as non-participants were somewhat older than participants. The 65% participation rate is however satisfactory in the cancer context. Second, the age distribution of our sample was skewed toward higher age, as it is mostly the case in cancer patient samples. Our model did however not violate regression assumptions, as homoscedasticity of regression residuals across the age continuum was confirmed and no evidence of extreme outliers was found, supporting the generalizability of our data. However inclusion of patients older than 75 years would have provided a fuller picture. Another limitation was the unequal distribution of patients receiving curative and palliative treatment. Although the assumption of homogeneous error variances across subgroups was met, this reduced the power of our moderator analyses. The variance explained by the interaction was small, but within the range usually expected for three-way interactions [32]. Finally, the simple dichotomy of curative vs. palliative treatment phase does not reflect the progress in cancer treatment toward gradual transition from curative to palliative care. The related decline of strict criteria to distinguish curative from palliative treatment phase involves a subjective evaluation of treatment phase by the attending physician. It may further explain the 8% of participating patients we had to exclude from analyses due to unclear treatment phase. The dichotomy does further not attend to possible differences in existential challenges between patients with early-stage and advanced cancer receiving treatment with curative intention according to their treating physician [57]. However, we chose to examine the psychological impact of physician-rated treatment intention rather than tumor stage because this may provide a closer approach to practical changes in communication about prognosis and treatment goals relevant to the perceived existential threat in the context of progressive disease.

In conclusion, the present study shows that existential distress in terms of demoralization is (a) frequent in cancer and (b) depends on a complex interaction of demographic and medical patient characteristics. One explanation to the latter is that existential challenges related to palliative treatment bring age- and gender-specific vulnerability and protective factors of existential distress into sharper relief. Moreover, these results provide a new perspective on the limited or inconsistent simple effects of palliative treatment, age, and gender on existential distress in previous research; future studies should consider that the effects of these variables depend on their sample-based distribution, which is to a large extent determined by the tumor site of the patients studied. Further improvement of knowledge about subgroup resources and vulnerabilities in the context of existential threat would provide valuable input for interventions focusing on the individual experience of existential challenges among cancer patients.

## References

[1] Kissane DW, Treece C, Breitbart W, McKeen NA, Chochinov HM (2009) Dignity, meaning and demoralization: Emerging paradigms in end-of-life-care. In: Chochinov HM, Breitbart W, editors. Handbook of psychiatry in palliative medicine. New York: Oxford University Press. pp. 324–340.

[2] Breitbart W, Poppito S, Rosenfeld B, Vickers AJ, Li Y, et al (2012) Pilot randomized controlled trial of individual meaning-centered psychotherapy for patients with advanced cancer. J Clin Oncol 30 ((12)) 1304–1309. 22370330 10.1200/JCO.2011.36.2517 PMC3646315

[3] Nissim R, Freeman E, Lo C, Zimmermann C, Gagliese L, et al (2012) Managing Cancer and Living Meaningfully (CALM): A qualitative study of a brief individual psychotherapy for individuals with advanced cancer. Palliat Med 26 ((5)) 713–721. 22042225 10.1177/0269216311425096

[4] Rodin G, Zimmermann C (2008) Psychoanalytic reflections on mortality: a reconsideration. J Am Acad Psychoanal Dyn Psychiatry 36 ((1)) 181–196. 18399753 10.1521/jaap.2008.36.1.181

[5] Chochinov HM (2006) Dying, dignity, and new horizons in palliative end-of-life care. CA Cancer J Clin 56 ((2)) 84–103. 16514136 10.3322/canjclin.56.2.84

[6] LeMay K, Wilson KG (2008) Treatment of existential distress in life threatening illness: A review of manualized interventions. Clin Psychol Rev 28 (3): 472–493. 17804130 10.1016/j.cpr.2007.07.013

[7] Clarke DM, Kissane DW (2002) Demoralization: its phenomenology and importance. Aust N Z J Psychiatry 36 (6): 733–742. 12406115 10.1046/j.1440-1614.2002.01086.x

[8] de Figueiredo JM (1993) Depression and demoralization: Phenomenological differences and research perspectives. Compr Psychiatry 34 (5): 308–311. 8306640 10.1016/0010-440x(93)90016-w

[9] Kissane DW, Clarke DM, Street AF (2001) Demoralization syndrome-a relevant psychiatric diagnosis for palliative care. J Palliat Care 17 (1): 12–21. 11324179

[10] Angelino AF, Treisman GJ (2001) Major depression and demoralization in cancer patients: diagnostic and treatment considerations. Support Care Cancer 9 (5): 344–349. 11497387 10.1007/s005200000195

[11] Connor MJ, Walton JA (2011) Demoralization and remoralization: a review of these constructs in the healthcare literature. Nurs Inq 18 (1): 2–11. 21281390 10.1111/j.1440-1800.2010.00501.x

[12] Clarke DM, Kissane DW, Trauer T, Smith GC (2005) Demoralization, anhedonia and grief in patients with severe physical illness. World Psychiatry 4 (2): 96–105. 16633525 PMC1414748

[13] Jacobsen J, Vanderwerker LC, Block SD, Friedlander RJ, Maciejewski PK, et al (2006) Depression and demoralization as distinct syndromes: Preliminary data from a cohort of advanced cancer patients. Indian J Palliat Care 12 (1): 8–15.

[14] Kissane DW, Wein S, Love A, Lee XQ, Kee PL, et al (2004) The Demoralization Scale: a report of its development and preliminary validation. J Palliat Care 20 (4): 269–276. 15690829

[15] Grassi L, Sabato S, Rossi E, Biancosino B, Marmai L (2005) Use of the diagnostic criteria for psychosomatic research in oncology. Psychother Psychosom 74 (2): 100–107. 15741759 10.1159/000083168

[16] Vehling S, Lehmann C, Oechsle K, Bokemeyer C, Krüll A, et al (2012) Is advanced cancer associated with demoralization and lower global meaning? The role of tumor stage and physical problems in explaining existential distress in cancer patients. Psychooncology 21 (1): 54–63. 21061407 10.1002/pon.1866

[17] Mehnert A, Vehling S, Hoecker A, Lehmann C, Koch U (2011) Demoralization and depression in patients with advanced cancer: validation of the German version of the demoralization scale. J Pain Symptom Manage 42 (5): 768–776. 21592722 10.1016/j.jpainsymman.2011.02.013

[18] Grassi L, Rossi E, Sabato S, Cruciani G, Zambelli M (2004) Diagnostic criteria for psychosomatic research and psychosocial variables in breast cancer patients. Psychosomatics 45 (6): 483–491. 15546825 10.1176/appi.psy.45.6.483

[19] Cockram CA, Doros G, Figueiredo JM de (2009) Diagnosis and measurement of subjective incompetence: the clinical hallmark of demoralization. Psychother Psychosom 78 (6): 342–345. 19713728 10.1159/000235737

[20] Lee CY, Fang CK, Yang YC, Liu CL, Leu YS, et al (2012) Demoralization syndrome among cancer outpatients in Taiwan. Support Care Cancer 20 (10): 2259–2267. 22120003 10.1007/s00520-011-1332-4

[21] Chochinov HM, Hassard T, McClement S, Hack T, Kristjanson LJ, et al (2009) The landscape of distress in the terminally ill. J Pain Symptom Manage 38 (5): 641–649. 19713069 10.1016/j.jpainsymman.2009.04.021

[22] Kissane DW (2012) The relief of existential suffering. Arch Intern Med 172 (19): 1501–1505. 22945389 10.1001/archinternmed.2012.3633

[23] Boscaglia N, Clarke DM (2007) Sense of coherence as a protective factor for demoralisation in women with a recent diagnosis of gynaecological cancer. Psychooncology 16 (3): 189–195. 16869021 10.1002/pon.1044

[24] Scheier MF (2006) Really, disease doesn′t matter? A commentary on correlates of depressive symptoms in women treated for early-stage breast cancer. J Clin Oncol 24 (16): 2407–2408. 16651643 10.1200/JCO.2005.05.5244

[25] Mehnert A, Koch U, Schulz H, Wegscheider K, Weis J, et al (2012) Prevalence of mental disorders, psychosocial distress and need for psychosocial support in cancer patients-study protocol of an epidemiological multi-center study. BMC Psychiatry 12 (1): 70. 22747671 10.1186/1471-244X-12-70 PMC3434016

[26] Mehnert A, Lehmann C, Cao P, Koch U (2006) Die Erfassung psychosozialer Belastungen und Ressourcen in der Onkologie-Ein Literaturüberblick zu Screeningmethoden und Entwicklungstrends. Psychother Psychosom Med Psychol 56 (12): 462–479. 17160791 10.1055/s-2006-951828

[27] Revenson TA, Schiaffino KM, Majerovitz SD, Gibofsky A (1991) Social support as a double-edged sword: the relation of positive and problematic support to depression among rheumatoid arthritis patients. Soc Sci Med 33 (7): 807–813. 1948172 10.1016/0277-9536(91)90385-p

[28] Ullrich A, Mehnert A (2010) Psychometrische Evaluation und Validierung einer 8-Item Kurzversion der Skalen zur Sozialen Unterstützung bei Krankheit (SSUK) bei Krebspatienten. Klinische Diagnostik und Evaluation 3: 359–381.

[29] Lowe B, Kroenke K, Herzog W, Grafe K (2004) Measuring depression outcome with a brief self-report instrument: sensitivity to change of the Patient Health Questionnaire (PHQ-9). J Affect Disord 81 (1): 61–66. 15183601 10.1016/S0165-0327(03)00198-8

[30] Aiken LS, West SG (1991) Multiple regression: Testing and interpreting interactions. Newbury Park: Sage Publications

[31] Fox J (2008) Applied regression analysis and generalized linear models. Los Angeles: Sage. 665 p

[32] Whisman MA, McClelland GH (2005) Designing, testing, and interpreting interactions and moderator effects in family research. J Fam Psychol 19 (1): 111–120. 15796657 10.1037/0893-3200.19.1.111

[33] Dawson JF, Richter AW (2006) Probing three-way interactions in moderated multiple regression: development and application of a slope difference test. J Appl Psychol 91 (4): 917–926. 16834514 10.1037/0021-9010.91.4.917

[34] Alexander RA, DeShon RP (1994) Effect of error variance heterogeneity on the power of tests for regression slope differences. Psychol Bull 115 (2): 308–314.

[35] Aguinis H, Pierce CA (1998) Heterogeneity of error variance and the assessment of moderating effects of categorical variables: A conceptual review. ORM 1 (3): 296–314.

[36] Jones JM, Huggins MA, Rydall AC, Rodin GM (2003) Symptomatic distress, hopelessness, and the desire for hastened death in hospitalized cancer patients. J Psychosom Res 55 (5): 411–418. 14581095 10.1016/s0022-3999(03)00526-9

[37] Mosher CE, Danoff-Burg S (2005) A review of age differences in psychological adjustment to breast cancer. J Psychosoc Oncol 23 (2–3): 101–114. 16492654 10.1300/j077v23n02_07

[38] Arden-Close E, Gidron Y, Moss-Morris R (2008) Psychological distress and its correlates in ovarian cancer: a systematic review. Psychooncology 17 (11): 1061–1072. 18561287 10.1002/pon.1363

[39] Mehnert A, Lehmann C, Graefen M, Huland H, Koch U (2010) Depression, anxiety, post-traumatic stress disorder and health-related quality of life and its association with social support in ambulatory prostate cancer patients. Eur J Cancer Care 19 (6): 736–745. 10.1111/j.1365-2354.2009.01117.x 19832893

[40] Rosenfeld B, Roth AJ, Gandhi S, Penson D (2004) Differences in health-related quality of life of prostate cancer patients based on stage of cancer. Psychooncology 13 (11): 800–807. 15386638 10.1002/pon.797

[41] Nelson CJ, Weinberger MI, Balk E, Holland J, Breitbart W, et al (2009) The chronology of distress, anxiety, and depression in older prostate cancer patients. Oncologist 14 (9): 891–899. 19738000 10.1634/theoncologist.2009-0059 PMC2881474

[42] Miller S, Lo C, Gagliese L, Hales S, Rydall A, et al (2011) Patterns of depression in cancer patients: an indirect test of gender-specific vulnerabilities to depression. Soc Psychiatry Psychiatr Epidemiol 46 (8): 767–774. 20574846 10.1007/s00127-010-0246-7

[43] Giese-Davis J, Waller A, Carlson LE, Groff SL, Zhong L, et al (2012) Screening for distress, the 6th vital sign: common problems in cancer outpatients over one year in usual care: associations with marital status, sex, and age. BMC Cancer 12 (1): 441. 23031647 10.1186/1471-2407-12-441 PMC3528655

[44] Blank TO, Bellizzi KM (2008) A gerontologic perspective on cancer and aging. Cancer 112: 2569–2576. 18428204 10.1002/cncr.23444

[45] Hagedoorn M, Sanderman R, Bolks HN, Tuinstra J, Coyne JC (2008) Distress in couples coping with cancer: a meta-analysis and critical review of role and gender effects. Psychol Bull 134 (1): 1–30. 18193993 10.1037/0033-2909.134.1.1

[46] Kuehner C (2003) Gender differences in unipolar depression: an update of epidemiological findings and possible explanations. Acta Psychiatr Scand 108 (3): 163–174. 12890270 10.1034/j.1600-0447.2003.00204.x

[47] Nolen-Hoeksema S (2012) Emotion regulation and psychopathology: The role of gender. Annu Rev Clin Psychol 8 (1): 161–187. 22035243 10.1146/annurev-clinpsy-032511-143109

[48] Siegel K, Gluhoski V, Gorey E (1999) Age-related distress among young women with breast cancer. J Psychosoc Oncol 17 (1): 1–20.

[49] Sachs E, Kolva E, Pessin H, Rosenfeld B, Breitbart W (2012) On sinking and swimming: The dialectic of hope, hopelessness, and acceptance in terminal cancer. Am J Hosp Palliat Care 10.1177/1049909112445371 PMC4972334 22556280

[50] Tamres LK, Janicki D, Helgeson VS (2002) Sex differences in coping behavior: A meta-analytic review and an examination of relative coping. Pers Soc Psychol Rev 6 (1): 2–30.

[51] Lo C, Lin J, Gagliese L, Zimmermann C, Mikulincer M, et al (2010) Age and depression in patients with metastatic cancer: The protective effects of attachment security and spiritual wellbeing. Ageing Soc 30 (02): 325.

[52] Vehling S, Lehmann C, Oechsle K, Bokemeyer C, Krull A, et al (2011) Global meaning and meaning-related life attitudes: exploring their role in predicting depression, anxiety, and demoralization in cancer patients. Support Care Cancer 19 (4): 513–520. 20306275 10.1007/s00520-010-0845-6

[53] Rodin G, Lo C, Mikulincer M, Donner A, Gagliese L, et al (2009) Pathways to distress: the multiple determinants of depression, hopelessness, and the desire for hastened death in metastatic cancer patients. Soc Sci Med 68 (3): 562–569. 19059687 10.1016/j.socscimed.2008.10.037

[54] Monforte-Royo C, Villavicencio-Chávez C, Tomás-Sábado J, Mahtani-Chugani V, Balaguer A, et al (2012) What lies behind the wish to hasten death? A systematic review and meta-ethnography from the perspective of patients. PLoS ONE 7 (5): e37117. 22606338 10.1371/journal.pone.0037117 PMC3351420

[55] Brothers BM, Andersen BL (2009) Hopelessness as a predictor of depressive symptoms for breast cancer patients coping with recurrence. Psychooncology 18 (3): 267–275. 18702065 10.1002/pon.1394 PMC2743157

[56] Jacobsen JC, Maytal G, Stern TA (2007) Demoralization in medical practice. Prim Care Companion J Clin Psychiatry 9 (2): 139–143. 17607336 10.4088/pcc.v09n0208 PMC1896303

[57] Ryan D, Gallagher P, Wright S, Cassidy E (2012) Methodological challenges in researching psychological distress and psychiatric morbidity among patients with advanced cancer: What does the literature (not) tell us. Palliat Med 26 (2): 162–177. 21562030 10.1177/0269216311399663

